# Isolation of methyl caffeate and flacourtin from *Flacourtia jangomas* with comprehensive *in-vitro* and *in-vivo* pharmacological evaluation

**DOI:** 10.1016/j.heliyon.2024.e40445

**Published:** 2024-11-16

**Authors:** Sadia Afreen Chowdhury, Rajib Das, Md. Ariful Haq, A.T.M. Zafrul Azam, Choudhury Mahmood Hasan, Monira Ahsan

**Affiliations:** a Department of Pharmacy, Stamford University Bangladesh, Bangladesh; b Department of Clinical Pharmacy and Pharmacology, Faculty of Pharmacy, University of Dhaka, Dhaka, 1000, Bangladesh; c Pharmaceutical Sciences Research Division, Bangladesh Council of Scientific and Industrial Research (BCSIR), Dhaka, 1205, Bangladesh; d Department of Pharmaceutical Chemistry, Faculty of Pharmacy, University of Dhaka, Dhaka, 1000, Bangladesh

**Keywords:** *Flacourtia jangomas*, Antioxidant, Cytotoxicity, Thrombolysis, Antimicrobial, Analgesic

## Abstract

**Background:**

*Flacourtia jangomas,* the Indian Coffee Plum or Indian Plum, is a medicinal plant found in Bangladesh and South Asia. Renowned for its potential as a source of bioactive compounds, this plant has garnered attention for its diverse therapeutic applications. This study aims to isolate phytochemicals and investigate the biological properties of methanol extracts from the bark of *Flacourtia jangomas.*

**Material and methods:**

The dried coarse plant powder was extracted with methanol and dried with a rotary evaporator. Then, the plant extract was subjected to phytochemical screening using various test reagents. Furthermore, extracts were investigated for isolating and characterizing chemical compounds and some of their biological activities.

**Results:**

The chloroform fraction of the methanolic extract of Flacourtia jangomas (MEFJ) yielded methyl caffeate and flacourtin, the first ever reported from this plant. Secondary metabolites were found via phytochemical screening, and total phenolic content was assessed. MEFJ was compared to BHT in the DPPH experiment for antioxidant potential. The brine shrimp lethality assay showed MEFJ's greater cytotoxicity than vincristine sulfate. Compared to streptokinase, increasing concentration increased thrombolytic activity and clot lysis. Compared to ciprofloxacin, F. jangomas did not exhibit substantial antibacterial activity (P < 0.001). The antifungal activity is not significant compared to Griseofulvin under the same conditions (P < 0.001). MEFJ stabilized membranes better than diclofenac sodium. The 400 mg/kg group inhibited acetic acid-induced analgesia by 70.32 %, but the control group did not. MEFJ at 200 mg/kg relieved pain better than the reference drug and 400 mg/kg in the hot plate test. This indicates the need for additional research.

**Conclusion:**

*Flacourtia jangomas is a potential candidate for bioactive compounds, and further studies on the* isolation and characterization of its bioactive compounds are highly required.

## 1 Introduction

Medicinal plants are an essential source of raw materials for drug discovery because they contain a wide range of bioactive chemicals, such as alkaloids, flavonoids, phenolics, tannins, glycosides, and volatile oils. These compounds have much promise to have pharmacological effects and can be used to treat diseases. Researchers keep isolating and naming bioactive chemicals from medicinal plants, which add to our knowledge and give us more tools for making new drugs. So, medicinal plants are essential because they provide a valuable source of bioactive compounds that can be studied for their healing effects [1]. The World Health Organization (WHO) recently said that herbal medicines are used by about 80 % of people around the world as part of their primary health care. Approximately 450–500 plants grown in or found in Bangladesh are known for their medicinal properties [2]. *Flacourtia jangomas* is a medicinal plant native to Bangladesh and other South Asian regions*.* It is also known as Indian Coffee Plum or Indian Plum. It is often called Lukluki, Tokroi, Paniamala, or Paniala in Bangladesh. It is popular in Sylhet, Chittagong Hill-Tracts, and Cox's Bazar [3,4]. It's a tiny deciduous tree that typically reaches heights of 6–10 m but can get as tall as 14 m in rare cases. A spreading crown gives it a thick, bushy appearance. The leaves are simple, alternating, oval, and serrated around the edges. Bark ranges from pale brown to copper red to pinkish buff and peels off in thin, lenticelled lamels. White to greenish, with 4 or 5 ovate triangular petals and a honey scent, the dioecious flowers appear before or alongside the new foliage [5]. New, gorgeously green leaves and flowers bloom simultaneously from December to April [6]. Significant to *Flacourtia jangomas* is the plant's fruit. It's a tiny berry, usually about a centimeter or two across. The fruit starts green but ripens to a deep purple or even black. The flavor is sour and tangy. The fruits can be eaten fresh, cooked into other dishes, or preserved in various ways [7].

*Flacourtia jangomas* has been used for centuries as a medicinal herb. The plant's bark, leaves, and fruits are all used for their therapeutic value [8]. It has been believed to have beneficial effects against free radicals, inflammation, germs, fungi, and pain. Extracts from the plant have been researched for their possible efficacy in treating various problems, including those affecting the digestive, respiratory, and skin [9,10]. The plant's antioxidant and antibacterial properties originate from these bioactive components. *Flacourtia jangomas* are utilized for a variety of therapeutic purposes in alternative medicine. Diarrhea, dysentery, and fever can all be treated with the bark. Infections, wounds, and skin inflammation are treated topically with the leaves. The fruit is consumed for its refreshing taste and purported ability to ease stomach upset [6,11].

In recent years, studies have focused on isolating cytotoxic and antibacterial components from various plants, as these properties are valuable for medicinal use. The rapid proliferation of drug-resistant microorganisms has driven research into new antibacterial agents from plant extracts, aiming to identify novel chemical structures. Antimicrobial compounds from plants may offer substantial clinical benefits for treating resistant microbial strains by hindering bacterial growth or killing germs through different mechanisms than current antimicrobials [12]. Cytotoxic studies can also lead to further cancer drug development. *In-vivo* analgesic studies have been conducted to evaluate the effectiveness of plant extracts in living organisms, providing a more accurate assessment of their potential pain-relieving properties [13]. These studies help confirm the extract's therapeutic efficacy observed *in-vitro* and assess its safety and biological activity in a complex physiological environment, offering insights into the practical application of the extract for pain management.

This study aimed to isolate and identify bioactive compounds from the methanolic extract of *Flacourtia jangomas* bark, explicitly focusing on methyl caffeate and flacourtin. Additionally, the study sought to evaluate the extract's antioxidant, cytotoxic, thrombolytic, antibacterial, antifungal, membrane stabilizing, and analgesic properties. Through comprehensive *in-vitro* and *in-vivo* assays, the study aimed to elucidate the therapeutic potential of these compounds and their contribution to the plant's medicinal value.

## 2 Materials and methods

### 2.1 Plant material and extract preparation

The bark of *Flacourtia jangomas* was collected in October 2022 from the hilly areas of Maulavi Bazar, Bangladesh. Then, the plant (Accession number: DUSH 10825) was precisely recognized by Dr. Mohammad Zashim Uddin, Professor, Department of Botany, Faculty of Biological Sciences, University of Dhaka. After being cut up, it spent two weeks drying in the sun. The dried plant was ground into a coarse powder and stored in sealed containers after a final drying in a drier at 40 °C for 1 h. The container was kept in a dark, dry, and cool area.

### 2.2 Extraction and isolation

The powdered plant materials were weighed a net of 5.0 kg before drying and milling. After drying and milling, the powder weighed 3.0 kg, and a net water loss of 60 % could be seen. The plant was ground (2080 g) and extracted with Methanol (3.5L) for 15 days in a big glass container. We repeated the technique twice for three days with the identical solvent system. Clear filtrate was obtained by decanting the extract through a cotton stopper and filter paper. The filtrate was collected and concentrated using a rotary evaporator to obtain methanol crude extract (approximately 32.0 gm). Then, 30 gm methanol crude extract was partitioned by following Kupchan's partitioning method repetitively thrice time with n-hexane (7.0 gm), chloroform (10.0 gm), ethyl acetate (10.0 gm) and aqueous (25.0 gm) portion to get extractives.

The chloroform soluble extractive (7.0 gm) was fractionated by Sephadex column chromatography. The column was eluted with n-hexane, chloroform, and methanol mixture of increasing polarities to get 142 fractions. **Compound 1 (2.6 mg)** was isolated from fraction no.-136 by preparative thin layer chromatography (PTLC) over silica gel (mobile phase: toluene-ethyl acetate-methanol; 40:50:10 with few drops of acetic acid; multiple developments; thickness of plates: 0.5 mm). **Compound 2 (14.2 mg)** was isolated from fraction no.-140 by washing the crystal with 50:50 chloroform and methanol mixture. Compound 1 was isolated through UV detection at a short (254 nm) wavelength. The remaining compounds on the TLC plate were visualized by long (366 nm) wavelength and by spraying vanillin/H_2_SO_4_ followed by heating.

### 2.3 Experimental procedure-general

^1^H spectra were acquired using the Ultra Shield Bruker DPX 600 NMR instrument, and the chemical shifts are reported in ppm concerning TMS or residual non-deutarated solvent signals.

### 2.4 Phytochemical screening

According to the procedure outlined by Auwal et al. [14], a methanol extract of *Flacourtia jangomas* was subjected to a preliminary phytochemical study. This technique has recently gained considerable popularity [15].

### 2.5 Antioxidant activity test

#### 2.5.1 Determination of total phenolic content in extracts

The total phenolic content of the preparations was determined spectrophotometrically using the Folin–Ciocalteau method, which utilized the Folin–Ciocalteau reagent as an oxidizing agent and gallic acid as a standard [16]. During sample preparation, 5 mg of the dried extract were collected and diluted to a concentration of 2 mg/mL in distilled water. 5 mL Folin-Ciocalteu reagent (10 times diluted with water) and 4 mL Na_2_CO_3_ (7.5 % w/v) solution were added to 1 mL (2 mg/mL) extract solution. At room temperature, 20 min were spent incubating the mixtures. After 20 min, the absorbance at 765 nm was measured using a UV-spectrophotometer, and the total phenols content of the sample was calculated using a standard curve constructed from acid solutions of varying concentrations of GAE (Gallic acid equivalent). The phenolic content of the sample was measured in milligrams of GAE per gram of extract. As a standard, gallic acid was used. Gallic acid solutions ranging in concentration from 500 to 1.9531 μg/mL were manufactured. A linear relationship was established by plotting the absorbance in the y ordinate against the concentration in the x ordinate, which was then utilized to determine the total phenolic content of the test materials.

#### 2.5.2 Free radical scavenging activity using the DPPH method

One of the main goals of this study was to test the *F. jangomas* plant's potential to scavenge free radicals in a lab using 1,1-diphenyl-2-picrylhydrazyl (DPPH), following Baliyan et al., 's 2022 approach [17]. To experiment, 2.0 mL of a methanol solution containing various concentrations of *F. jangomas* plant extract was combined with 3.0 mL of a methanol solution containing 20 μg/mL of DPPH. The FJ plant extract was prepared by measuring and dissolving a calculated amount of plant methanol extract in methanol to produce a mother solution with a concentration of 1000 μg/mL. This mother solution was subsequently serially diluted to achieve concentrations ranging from 500.5 to 977 μg/mL. Using a UV spectrophotometer, the antioxidant potential of the FJ plant extract was determined by comparing the degree of discoloration of the purple-colored methanol solution containing DPPH to the degree of discoloration caused by ascorbic acid. The DPPH radical, which has an unpaired electron, has a prominent absorption peak at 517 nm and appears purple. As the odd electron of the DPPH radical pairs with hydrogen from a free radical-scavenging antioxidant, resulting in the formation of reduced DPPH-H, the DPPH radical's molar absorptivity at 517 nm diminishes, causing the color to change from purple to yellow. The IC_50_ value expresses the concentration of the *F. jangomas* plant extract required to achieve a 50 % reduction in DPPH as a measure of the FJ extract's ability to neutralize DPPH radicals. In the experiment, tert-butyl-1-hydroxytoluene (BHT) served as a positive control. Inhibition of free radical DPPH in percent (I%) was calculated in equation (i) as follows-

(i) (I %) = (1 – Absorbance of sample/ Absorbance of blank) × 100

Where the absorbance of the blank is the absorbance of the control reaction (containing all reagents except the test material). Extract concentration providing 50 % inhibition (IC_50_) was calculated from the graph plotting inhibition percentage against extract concentration.

### 2.6 Cytotoxicity screening

The brine shrimp lethality bioassay was used to determine the cytotoxicity of compounds using the simple zoological organism *Artemia salina* as a practical screening indicator collected from I&V BIO Artemia Nauplii Center, Cox's Bazar, Bangladesh. These nauplii were tested for cytotoxicity using the method outlined by Banti et al. [18]. The brine shrimp eggs were incubated for 48 h in artificial seawater with a constant oxygen supply containing a 3.8 % NaCl solution until they developed into mature shrimp known as nauplii, by dissolving 38 gm of purified sodium chloride (NaCl) in 1L of distilled water, which was then filtered, a clear solution was created. The shrimp eggs were added to a small vessel of seawater, and after two days of hatching and maturing under a constant oxygen supply, the newly born shrimp were attracted to a lamp through a perforated dam. Using a Pasteur pipette, ten living shrimp were added to each test tube containing 5 mL of seawater. Each test sample was dissolved in 100 μL of purified dimethyl sulfoxide (DMSO) to produce a stock solution. In the first test container, 50 μL of this stock solution was mixed with 5 mL of seawater and ten shrimp nauplii, resulting in a final 400 μg/mL concentration. Consequently, a series of solutions with varying concentrations (ranging from 400 to 0.78125 μg/mL) were prepared by serial dilution. The positive control consisted of vincristine sulfate with an initial concentration of 20 μg/mL and a final concentration of 0.0390 μg/mL after DMSO dilution. The control groups consisted of 10 brine nauplii suspended in 5 mL of simulated seawater to which the positive control solutions were added. 30 μl of DMSO was added as a negative control to each of the three pre-marked glass containers containing 5 mL of simulated seawater and ten shrimp nauplii. After 24 h, containers were examined with a magnifying glass to determine the number of survivors. For every dilution, the percentage of mortality was computed. The statistical significance of the concentration-mortality data was determined by probit analysis and linear regression using a specialized program on an IBM-PC. The median lethal concentration (LC_50_) was used to convey the efficacy of the plant product or the concentration-mortality relationship.

### 2.7 Thrombolytic activity

A method described by Sikder et al. (2011) that used streptokinase (SK) as the reference standard was used to assess the thrombolytic activity of the plant extract [19]. In multiple vials, 10 mg of *Flacourtia jangomas* methanolic extracts were taken, and 1 mL of distilled water was added. Then, 5 mL of sterile, distilled water was added, and the commercially available, lyophilized Altepase (Streptokinase of Popular Pharma) of 1500,000 IU was carefully mixed in. From this suspension, 100 μl (30,000 IU) was taken to perform *in-vitro* thrombolysis. 1 mL of whole blood (n = 10) was placed in the previously weighed sterile Eppendorf tubes and allowed to develop clots. The healthy human volunteers (n = 10) had no history of oral contraceptives or anticoagulant medication. Healthy volunteers provided aliquots (5 mL) of venous blood, divided among ten pre-weighed sterile Eppendorf tubes (0.5 mL/tube), and incubated at 37 °C for 45 min. Each Eppendorf tube containing a clot was weighed again to estimate the clot weight (clot weight = weight of clot-containing tube - the weight of tube alone) after the serum had been entirely withdrawn without disrupting the clot.

Separately, 100 μl aqueous solutions of various extracts and the crude extracts were added to each Eppendorf tube containing a pre-weighed clot. The control eppendorf tubes received 100 μl of streptokinase (SK) as a positive control and 100 μl of distilled water as a negative non-thrombolytic control, respectively. All Eppendorf tubes were incubated for 90 min at 37 °C to check for clot lysis. The discharged fluid was taken out after incubation, and Eppendorf tubes were weighed once more to measure the difference in weight following clot disruption. The difference obtained in weight taken before and after clot lysis was expressed as a percentage of clot lysis in equation (ii) as shown below:

(ii) % clot lysis = (Weight of the clot after lysis / Weight of clot before lysis) × 100

### 2.8 Antimicrobial activity assessment

#### 2.8.1 Antibacterial activity

##### 2.8.1.1 Bacterial strains for testing

The Department of Microbiology at Stamford University Bangladesh provided pure cultures of Gram-positive bacteria, including *Bacillus cereus, Staphylococcus aureus*; Gram-negative bacteria, comprising *Escherichia coli*, *Vibrio cholera* and *Klebsiella pneumoniae.*

##### 2.8.1.2 Antibacterial susceptibility test

The antibacterial activity of MEFJ was evaluated using the disc diffusion technique [20], incorporating both Gram-positive and Gram-negative bacterial strains. MEFJ was dissolved in methanol solvents to attain 300, 500, and 700 μg/mL concentrations. Sterile filter paper discs (5.0 mm in diameter) were impregnated with predetermined concentrations of the test solutions using a micropipette. The test microorganisms were uniformly distributed onto the nutrient agar medium, and discs containing the test material were placed on the medium. A positive control comprised a standard antibiotic disc containing Ciprofloxacin at an equivalent concentration, while negative controls featured blank discs impregnated with solvents. The plates were incubated at 37 °C for 24 h to facilitate optimal microbial growth. The antibacterial agents impeded microbial proliferation, resulting in a well-defined zone of inhibition observed around the medium and quantifying the antibacterial effectiveness of the test agents involved measuring the diameter of the inhibition zone in millimeters. The procedure was replicated thrice, and the average results were analyzed.

#### 2.8.2 Antifungal susceptibility assessment

##### 2.8.2.1 Fungal strains

Pure cultures of fungi, namely *Penicillium chrysogenum, Aspergillus niger, Mucor hiemalis, and Saccharomyces cerevisiae*, were obtained from the Microbiology Department of Stamford University Bangladesh.

##### 2.8.2.2 Disc diffusion method

The antifungal activity of MEFJ was evaluated using the disc diffusion assay [21]. In this methodology, a solid agar medium was prepared in Petri dishes, followed by the uniform spreading of 1 mL culture of each fungus across the medium. Sterile filter paper discs, measuring 6 mm in diameter, were impregnated with diluted MEFJ (10 μl) and placed on each agar plate. MEFJ was tested at various concentrations (300, 500, 700 μg/mL) in methanol solvent. Subsequently, the plates were incubated for 24 h in the incubator. Griseofulvin-containing discs were positive controls for antifungal activity, while discs containing methanol were employed as negative controls. Following the incubation period, the antifungal activity was determined based on the size of the inhibition zone surrounding each disc, measured in millimeters [22].

### 2.9 Membrane stabilization assay

#### 2.9.1 Preparation of human red blood cell (HRBC) suspension

As documented in various studies, the membrane stabilization method utilizing HRBCs was employed to investigate anti-inflammatory properties [23]. Fresh blood was obtained from healthy donors who had abstained from NSAIDS for a minimum of two weeks before the experiment. A sterile Alsever solution was prepared using 2 % dextrose, 0.8 % sodium citrate, 0.05 % citric acid, and 0.42 % sodium chloride in water, which was then mixed in equal proportions with freshly collected human blood. Subsequent centrifugation of the blood at 3000 rpm for 10 min, followed by washing the packed cells three times with isosaline (0.85 %, pH 7.2), formed a 10 % suspension with isosaline. The volume of the reconstituted blood was measured [24].

#### 2.9.2 Heat-induced hemolysis

The underlying principle of this method revolves around the stability of the human red blood cell membrane under hypo-tonicity-induced hemolysis. The reaction mixture consisted of 0.15M phosphate buffer (1 mL, pH 7.4), 0.36 % hyposaline (2 mL), 10 % v/v HRBC suspension (0.5 mL), plant extracts (0.5 mL), and diclofenac sodium as a standard drug. The control group used distilled water instead of hyposaline to induce 100 % hemolysis. The incubation period was set at 37 °C for 30 min, followed by centrifugation. Hemoglobin content in the suspension was determined using a spectrophotometer at 560 nm. The formula employed to estimate the percentage of HRBC membrane hemolysis is in equation (iii) as follows:

(iii) % Hemolysis = (Optical density of Test sample / Optical density of Control) X 100

equation (iv) utilized for determining the percentage of HRBC membrane stabilization:

(iv) % Protection = 100 – [(Optical density of Test sample / Optical density of Control) X 100]

### 2.10 Screening of analgesic activity

The analgesic activity of the methanol extract of *F. jangomas* was screened using two widely used models: acetic acid-induced writhing and the hot-plate test.

#### 2.10.1 Acetic acid-induced writhing method

The analgesic activity of the extracts was tested in mice using an acetic acid-induced writhing model [25]. Five mice each were placed in the control, positive control, and test groups of the animals. Test samples were administered to the test animals at 200 and 400 mg/kg body weight. The positive control group was given the standard medication diclofenac sodium at 10 mg/kg body weight, while the vehicle control group was given 10 mL of 1 % DMSO in water. Diclofenac-Na was administered 15 min before the injection of 0.7 % acetic acid, although the test sample and vehicle were given oral doses 30 min before the injection. After a break of 5 min, the mice were monitored for a specific body contraction known as "writhing" over the following 10 min. Positive control (Diclofenac-Na), control, and test samples given orally 30 min before acetic acid injection were compared for writhing. If the sample has analgesic activity, the animal that received it will writhe less frequently than the control, meaning that the analgesic activity of the sample will prevent writhing. An example of a reference standard drug is Diclofenac-Na.

#### 2.10.2 Hot-plate test

Based on the method described by Hijazi et al., 2017, the hot plate test quantifies the response latencies [26]. The hot plate temperature in this experiment was kept at 50 ± 0.5 °C. Animals pretreated with DMSO (10 mL/kg, orally, 30 min before) as control, a methanol extract of *Flacourtia jangomas* (200 mg/kg and 400 mg/kg, orally, 30 min before), and morphine (5 mg/kg, intraperitoneally, 15 min before), which was utilized as a positive control group, were timed to see how they reacted. Animals were placed inside a hot plate chamber, and the latency time was measured as the interval between the animal's placement on the hot plate surface at zero degrees Celsius and its licking or jumping off to escape the heat. The index of antinociception, known as the latent duration of response, was established at the pretreatment stage. The test medication and standard should be administered 30, 60, and 90 min later to limit the harm to the animal's paws. Twenty seconds were chosen as the cutoff time [27].

### 2.11 Statistical analysis

All statistical analyses in this study were conducted using Microsoft Excel and IBM SPSS Version 26. Microsoft Excel was used for initial data organization and basic descriptive statistics, while IBM SPSS Version 26 facilitated the more advanced statistical tests and analyses. This combination of software ensured accurate and reliable statistical results, supporting the conclusions drawn regarding *F. jangomas's* different activities.

## 3 Results and discussion

### 3.1 Isolation and identification

**Fig. 1:**
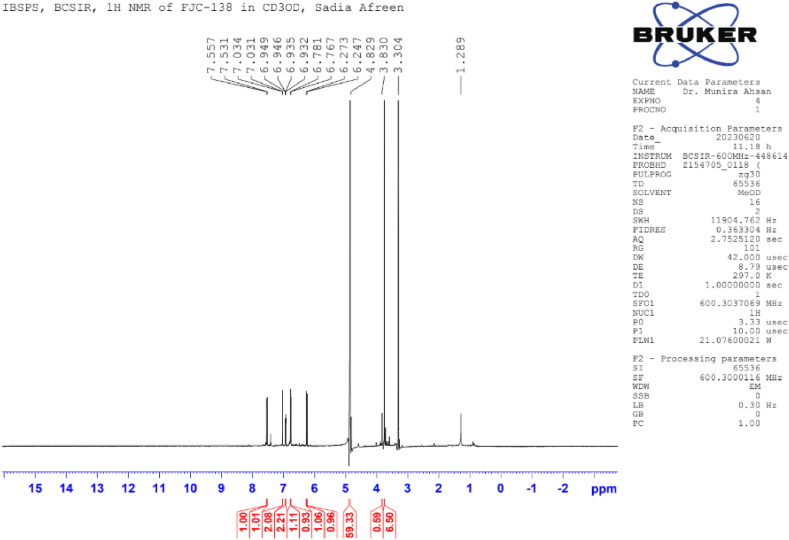
^1^H NMR spectroscopic Data (600 MHz, CD_3_OD) for Compound 1 (FJC-138).

**Fig. 2:**
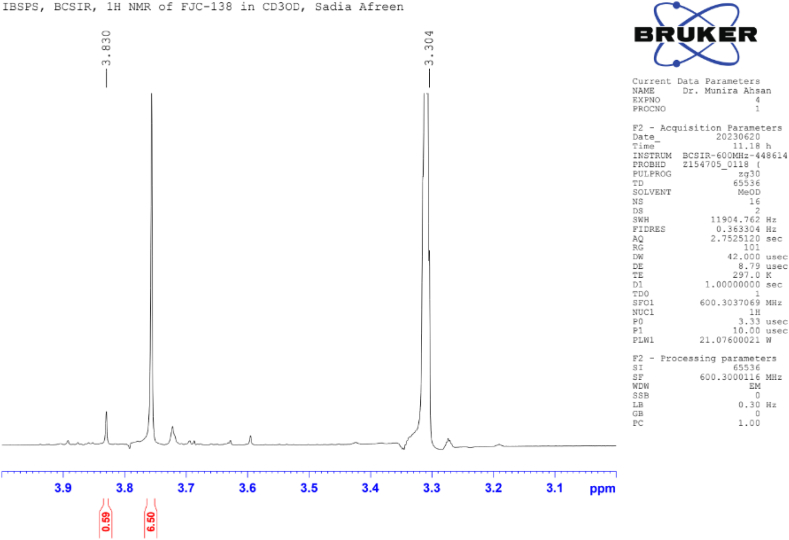
Partially Expanded ^1^H NMR spectroscopic Data (600 MHz, CD_3_OD) for Compound 1 (FJC-138).

**Fig. 3:**
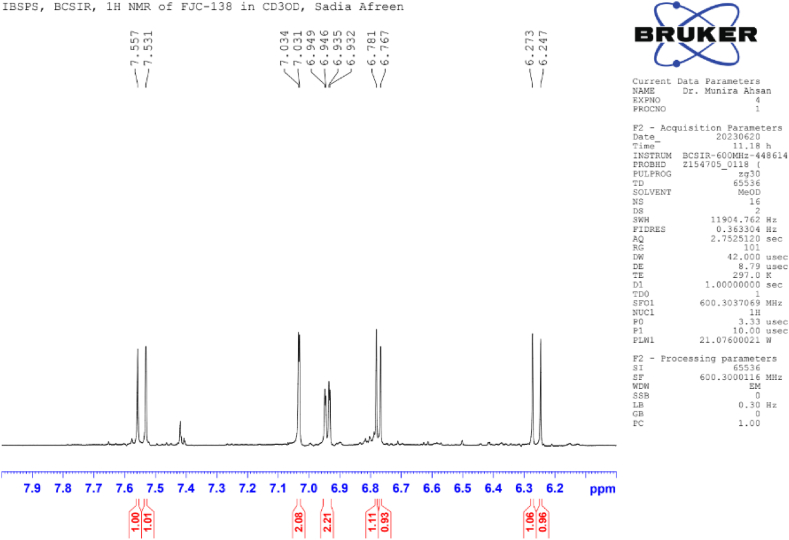
Partially Expanded ^1^H NMR spectroscopic Data (600 MHz, CD_3_OD) for Compound 1 (FJC-138).

**Methyl Caffeate (1)**: White crystal powder; ^1^H NMR (600 MHz, CD_3_OD): δ 7.03 (1H, dd, *J* = 8.4, 1.8 Hz, H-2), 6.77 (1H, d, *J* = 8.4 Hz, H-5), δ 6.94 (1H, dd, *J* = 8.4, 1.8 Hz, H-6), 6.26 (1H, d, *J* = 15.9 Hz, H-7), 7.55 (1H, d, *J* = 15.9 Hz, H-8), 3.76 (3H, s, OCH_3_) (Table 1) (Fig. 1, Fig. 2, Fig. 3).

**Table 1:** NMR spectroscopic Data (600 MHz, CD_3_OD) for Compound 1.

Position	Compound 1 (FJC-138) *δ*_H_	Methyl caffeate [26] *δ*_H_
2	7.03 dd (*J* = 1.8 Hz)	7.05 dd (*J* = 2.0 Hz)
5	6.77 d (*J* = 8.4 Hz)	6.79 d (*J* = 8.0 Hz)
6	6.94 dd (*J* = 8.4, 1.8 Hz)	6.95 dd (*J* = 8,0, 2.0 Hz)
7	6.26 d (*J* = 15.9 Hz)	6.27 d (*J* = 16 Hz)
8	7.55 d (*J* = 15.9 Hz)	7.55 d (*J* = 16 Hz)
OCH_3_	3.76 3H s	3.77 3H s

**Fig. 4:**
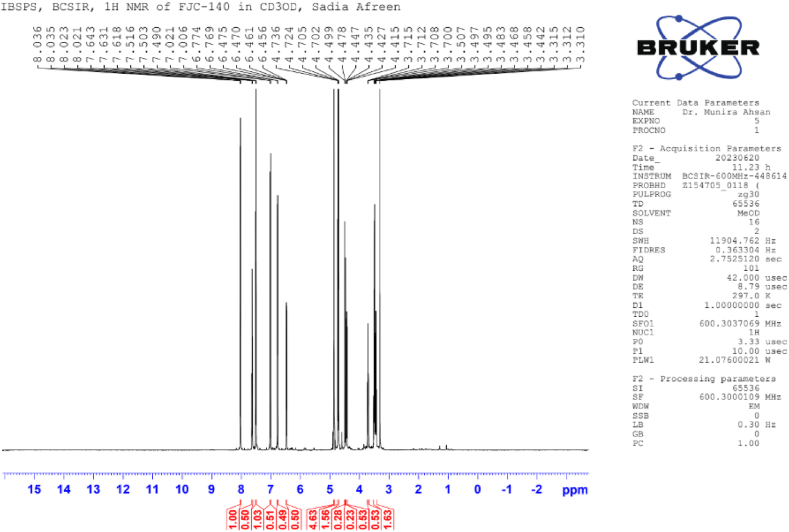
^1^H NMR spectroscopic Data (600 MHz, CD_3_OD) for Compound 2 (FJC-140).

**Fig. 5:**
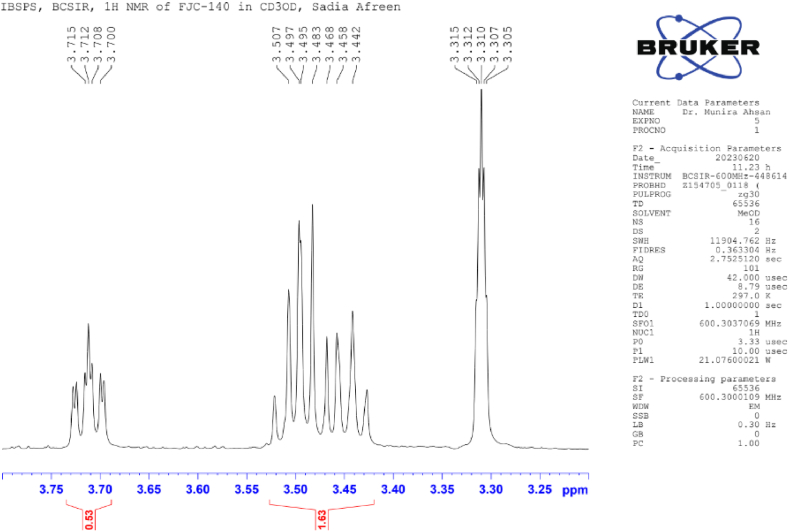
Partially Expanded ^1^H NMR spectroscopic Data (600 MHz, CD_3_OD) for Compound 2 (FJC-140).

**Fig. 6:**
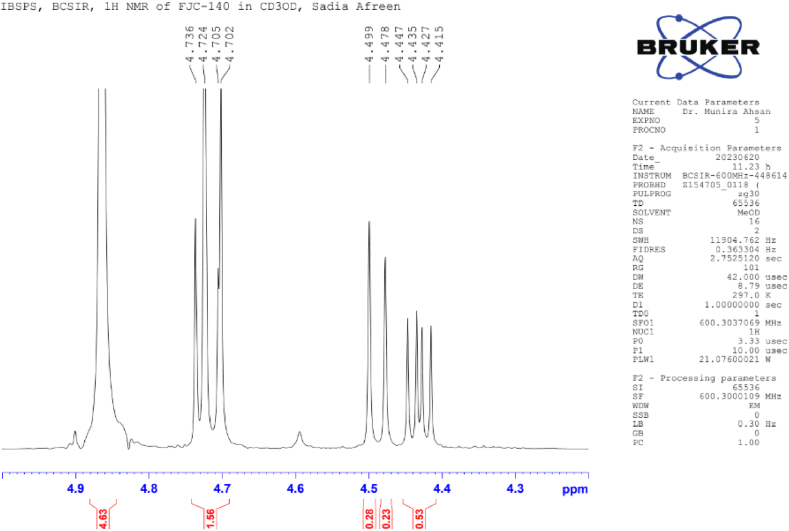
Partially Expanded ^1^H NMR spectroscopic Data (600 MHz, CD_3_OD) for Compound 2 (FJC-140).

**Fig. 7:**
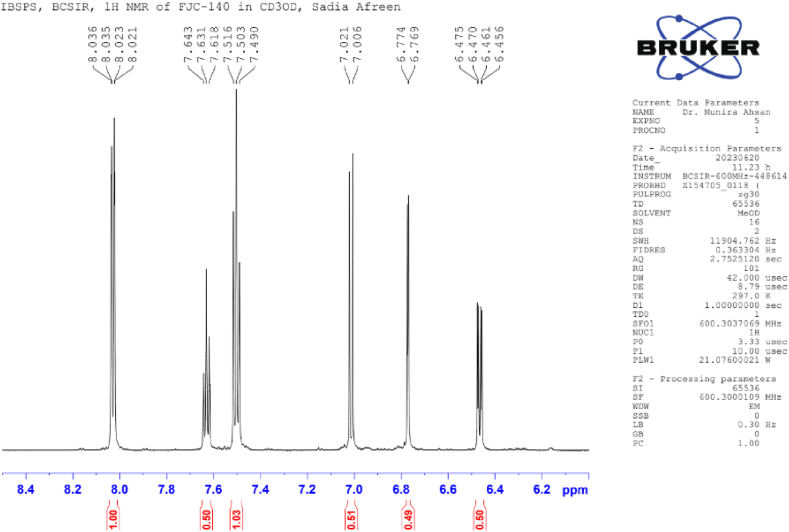
Partially Expanded ^1^H NMR spectroscopic Data (600 MHz, CD_3_OD) for Compound 2 (FJC-140).

**Flacourtin (2)**: White crystal; ^1^H NMR (600 MHz, CD_3_OD): δ 8.02 (2H, d, *J* = 7.6 Hz, H-2), 7.50 (2H, dd, *J* = 8.4, 7.6 Hz, H-3), δ 7.62 (1H, t, *J* = 7.4 Hz, H-4), 8.02 (2H, d, *J* = 7.6 Hz, H-6), 6.76 (1H, d, *J* = 2.8 Hz, H-2′), 7.00 (1H, d, *J* = 8.8 Hz, H-5′), 6.54 (1H, dd, *J* = 8.6, 3.0 Hz, H-6′), 4.70; 4.47 (3H, m), 4.30 (1H, s, OH-7′), 4.62 (1H, d, *J* = 4.85 Hz, H-1″), 3.48 (1H, m, H-2″, H-3″, H4″), 3.70 (1H, t, *J* = 11.8 Hz, H-6″), 4.42 (1H, dd, *J* = 11.8, 7.4 Hz, H-6″), 4.73 (1H, br s, OH-2″) (Table 2) (Fig. 4, Fig. 5, Fig. 6, Fig. 7).

**Figure:**
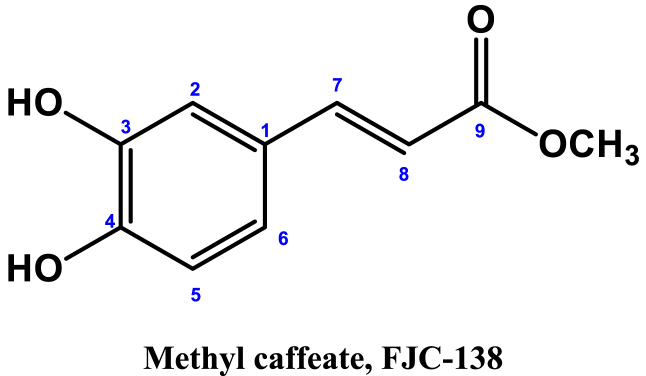

**Figure:**
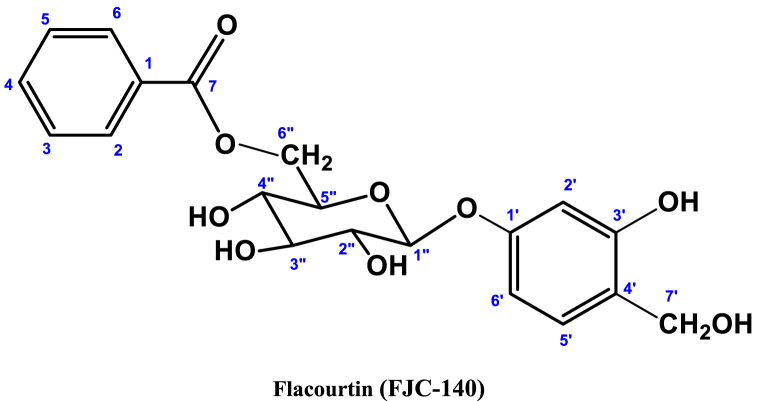

**Table 2:** NMR spectroscopic Data (600 MHz, CD_3_OD) for Compound 2.

Position	Compound 2 (FJC-140) *δ*_H_	Flacourtin [27] *δ*_H_
2	8.02 d (*J* = 7.6 Hz)	8.09 m (*J* = 7.45. 4.05 Hz)
3	7.50dd, *J* = 8.0, 7.6	7.56 dd, *J* = 8.0, 7.45
4	7.62tJ = 7.4	7.68 tJ = 8.0
5	7.50dd, *J* = 8.0, 7.6	7.56 dd, *J* = 8.0, 7.45
6	8.02 d (*J* = 7.6 Hz)	8.09 m (*J* = 7.45. 4.05 Hz)
2′	6.76 d(*J* = 2.8)	6.82 d(*J* = 3.05)
5′	7.00 d(*J* = 8.8)	7.11 d(*J* = 8.5)
6′	6.54 dd (*J* = 8.6, 3.0)	6.54 dd (*J* = 8.5,3.1)
7′	4.70; 4.47	4.75; 4.43
OH-7′	4.30	4.30
1”	4.62 d(*J* = 4,85)	4.77 d(*J* = 4,85)
2”	3.48m	3.55 m
3”	3.48*m*	3.55 *m*,
4”	3.48*m*	3.55 (1H, *m*)
5”	3.70t (*J* = 7.4)	3.83 dt (*J* = 9.55; 9.35
6”	4.70 d (*J* = 11.8) 4.42 dd (*J* = 11.8, 7.4) *m*	4.76 *m,* 4.45 m
OH-2”	4.73 br s	4.60

After repeated chromatographic separation and purification, a chloroform extract of the bark of *Flacourtia jangomas* provided two compounds (1 & 2), the structure of which was determined by extensive NMR spectral analysis and by comparing their spectral data with previously reported values.

^1^H NMR spectrum of compound 1 showed signal for three aromatic protons at δ 7.03, 6.94 and 6.77 ppm, and two conjugated olefinic groups (J = 15.6 Hz) at δ 6.26 and 7.55 (Table 1) (Fig. 3). Three protons singlet at δ 3.76 indicate the presence of methyl ester moieties (Fig. 2). These data were identified as methyl caffeate while comparing with the published ^1^H NMR data [28].

Compound 2 revealed the characteristic pattern of a phenolic ester glycoside flacourtin while analyzing 1H NMR spectra in CD3OD in a 600 MHz instrument compared with the published ^1^H NMR data of standard flacourtin [29]. The appearance of one proton doublet centered at *δ* 4.70 (*J* = 11.8 Hz) indicates the anomeric proton of glucose, which is axial to the pyranose ring [6,7]. Peaks clustered at *δ* 3.48–4.47 indicated the presence of sugar moiety (Table 2). The aromatic regions showed the presence of eight protons where the five protons appeared between *δ* 7.62 to *δ* 8.02 ppm, indicating the presence of benzoic acid residue of flacourtin (Fig. 7). Three methine protons were confirmed by multiplet at *δ* 3.48 ppm on the glucose moiety (Fig. 5).

### 3.2 Phytochemical screening

Form this screening test, it was identified that reducing Sugar, Tannins, saponins, alkaloids, glycosides, and flavonoids were present, whereas, carbohydratesand steroids were absent in MEFJ (Table 3).

**Table 3:** Qualitative phytochemical analysis of MEFJ.

Solvent	Steroids	Reducing Sugar	Tannins	Saponins	Alkaloids	Flavonoids	Carbohydrates	Glycosides
MEFJ	–	+	+	+	+	+	–	+

### 3.3 Antioxidant activity test

#### 3.3.1 Determination of total phenolic in extracts

**Fig. 8:**
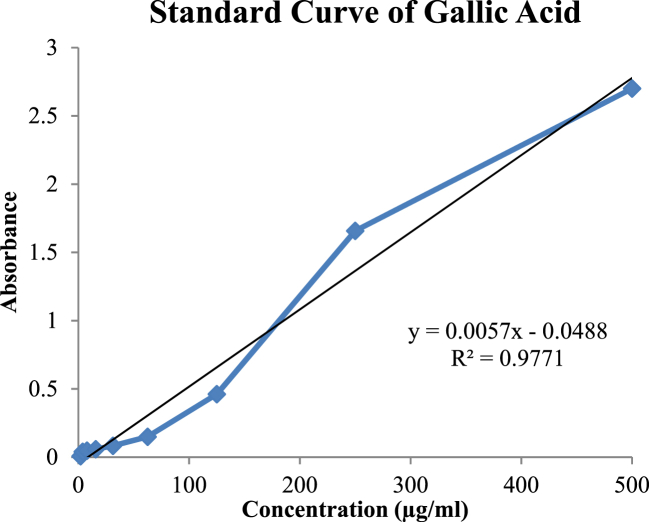
Standard curve of gallic acid for total phenolic determination.

The amount of TPC was determined by the Folin-Ciocalteu method. Gallic acid was used as a standard and total phenols were expressed as mg/g GAE using standard curve equation: *Y* = 0.0057X-0.0488, R^2^ = 0.9771, where Y is absorbance at 760 nm and X is TPC in *Flacourtia jangomas* extract (Fig. 8). The total phenolic content measured in F. *jangomas* extract was 62.24 mg gallic acid equivalents/g extract. Total phenolic contents measured by Folin-Ciocalteu method shown in Table 4.

**Table 4:** Total Phenolic Content of methanol extract of *F. jangomas* Plant.

Plant	Sample code	Absorbance	Average	Total phenolic content (mg of GAE/gm of extractive)
***FJ plant***	MEFJ	0.387	0.360	62.24
		0.335		
		0.359		

#### 3.3.2 Radical scavenging activity using DPPH method

**Fig. 9:**
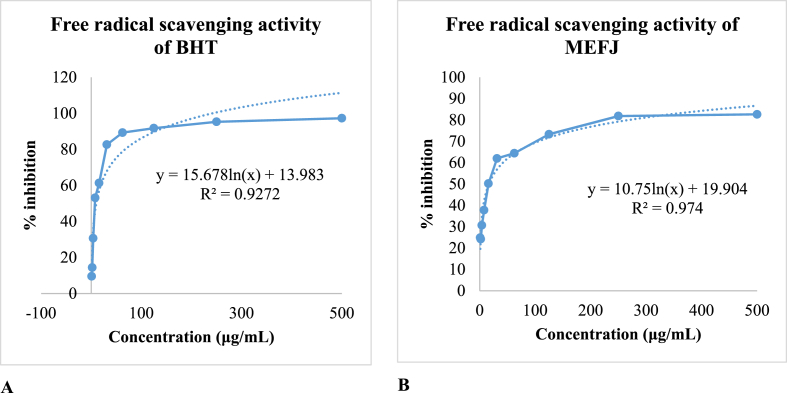
The antioxidant potential (IC50 value) of tert-butyl-1-hydroxytoluene (BHT) (A) and the methanol-soluble extract of F. sangomas (B) was observed with DPPH.

The antioxidant potential of the extract (IC_50_ value) of methanol extract of *F. jangomas* leaves in the DPPH method have been compared to the antioxidant potential of tert-butyl1-hydroxytoluene (BHT) (IC_50_ value) and the IC_50_ value has been found 16.4385 μg/mL for the antioxidant potential for methanol soluble extract of *F. jangomas* and IC_50_ value for the antioxidant potential for BHT was found 9.9463μg/mL. The summary of the IC_50_ values of the test sample and the tert-butyl-1-hydroxytoluene (BHT) have been presented in Fig. 9.

### 3.4 Cytotoxicity screening

**Fig. 10:**
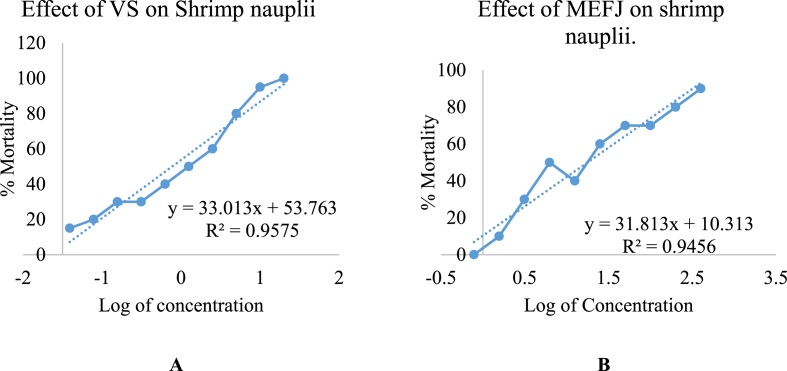
Plot of % mortality and predicted regression line of VS (A) and MEFJ (B).

In the context of the current investigation into bioactivity, all crude extracts yielded affirmative outcomes, signifying the biological activity of the test samples. Divergent mortality rates were observed for each test sample across varying concentrations. The log of concentration versus percent mortality was graphically represented for all test samples, revealing an approximately linear correlation. Subsequently, the median lethal concentration (LC_50_), denoting the concentration at which 50 % mortality of brine shrimp nauplii occurred, was determined based on the graphical representations. The LC_50_ values for the test samples after 24 h were derived by plotting the percentage of deceased shrimp against the logarithm of sample concentration (toxicant concentration), and the best-fit line was discerned through regression analysis of the curve data. Vincristine sulfate (VS) served as the positive control, and its LC_50_ was determined to be 0.769 μg/mL. Comparative analysis with the positive control revealed significant mortality, and the LC_50_ values of the various extracts were juxtaposed against this benchmark. Notably, the methanol extract derived from the entire plant of *F. jangomas* exhibited the most pronounced lethality, with an LC_50_ value of 1.247 μg/mL presented in Fig. 10 (Table 5).

**Table 5:** LC_50_ values of the test samples and standard.

Test samples	Regression line	R^2^	LC_50_ (μg/mL)
Standard	y = 33.01x + 53.77	0.957	0.769
MEFJ	y = 31.813x + 10.313	0.9456	1.247

### 3.5 Thrombolytic activity

As part of the exploration of cardio-protective pharmaceuticals derived from natural sources, the extractives of *F. jangomas* were evaluated for thrombolytic activity, and the findings are presented in Table 6. A total of 100 μl of Streptokinase (SK), constituting a positive control (30,000 IU of Streptokinase from Popular Pharma), was introduced to the clots, followed by an incubation period of 90 min at 37 °C, resulting in clot lysis. Conversely, distilled water served as the negative control, displaying a minimal percentage (16.67 %) of clot lysis. The mean difference in clot lysis percentage between the positive and negative controls was determined to be highly significant. In this preliminary investigation, the Streptokinase and methanol extract of *F. jangomas* demonstrated thrombolytic activities of 50.97 % and 82.58 %, respectively.

**Table 6:** Thrombolytic activity (in terms of % of clot lysis) of the extractives of *FJ.*

Plant Name	Weight of empty tube, W_1_ g	Weight of tube with clot before disruption, W_2_ g	Weight of tube with clot after disruption, W_3_ g	Weight of clot before lysis, W_4_ = (W_2_-W_1_) g	Weight of lysed clot, W_5_ = (W_2_-W_3_) g	% of lysis (W_5_/W_4_) × 100 %
Blank	0.7928	1.1743	0.8564	0.3815	0.0636	16.67
SK	0.7887	1.1736	0.9849	0.3849	0.1962	50.97
MEFJ	0.7822	1.1371	1.0753	0.3549	0.2931	82.58

### 3.6 Antimicrobial activity

#### 3.6.1 Antibacterial susceptibility test

**Fig. 11:**
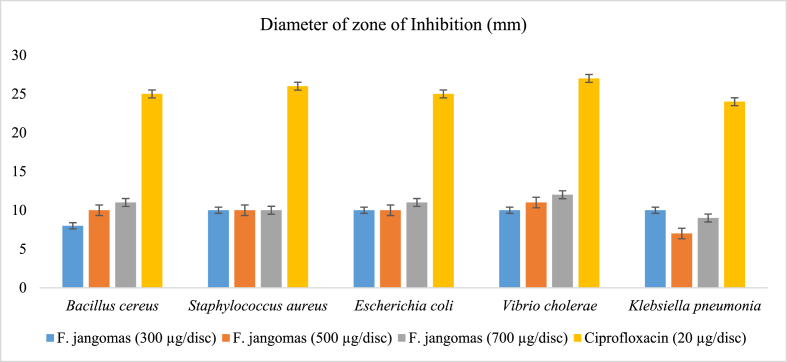
Diameter of zone of inhibition of *F. jangomas* (300, 500, and 700 μg/disc) against gram positive and gram negative bacterial strains.

The methanol-soluble extract derived from *F. jangomas* manifests mild inhibitory effects on bacterial growth, as evidenced by a range of zones of inhibition spanning from 8 mm to 12 mm. Notably, the maximum zone of inhibition was observed against *Vibrio cholerae*, measuring 12 mm, followed by 11 mm against both *Bacillus cereus* and *Escherichia coli*, all observed at a concentration of 700 μg/disc. Ciprofloxacin, employed as the standard drug, exhibited robust antibacterial activity, registering a zone of inhibition starting from 27 mm against *Vibrio cholerae*. The inhibitory effects on microbial growth are detailed in Table 7 and Fig. 11. In a one-way ANOVA, there was a significant difference (P < 0.0001) in the zone of inhibition across all bacterial strains for each group. A subsequent post hoc analysis using the Dunnett test revealed significant differences (P < 0.001) between all three concentrations of *F. jangomas* (300, 500, 700 μg/disc) and the standard ciprofloxacin (20 μg/disc) for all bacterial strains. These results suggest that F. jangomas may not possess significant antibacterial activity against these bacterial strains.

**Table 7:** Diameter of zone of inhibition of methanol extract of *F. jangomas*.

Test organisms	Diameter of Zone of Inhibition (mm)			
	*F. jangomas* (300 μg/disc)	*F. jangomas* (500 μg/disc)	*F. jangomas* (700 μg/disc)	Ciprofloxacin (20 μg/disc)
**Gram Positive Bacteria**				
*Bacillus cereus*	08	10	11	25
*Staphylococcus aureus*	10	10	10	26
**Gram Negative Bacteria**				
*Escherichia coli*	10	10	11	25
*Vibrio cholerae*	10	11	12	27
*Klebsiella pneumonia*	10	07	09	24

#### 3.6.2 Antifungal susceptibility test

The methanol-soluble extract derived from *F. jangomas* exhibits a moderate ability to inhibit the growth of microorganisms, as evidenced by the presence of inhibition zones of between 6 mm and 15 mm. The most remarkable result is the largest zone of inhibition found against *Mucor hiemalis,* reaching 15 mm, with 12 mm zones recorded against both *Saccharomyces cerevisiae* and *Aspergillus niger*, both at a dose of 700 μg/disc. On the other hand, Griseofulvin, used as the standard medicine, demonstrates notable antifungal properties by producing a 21 mm area where the growth of *Mucor hiemalis* is prevented. The specific impacts on the development of microorganisms are explained in detail in Table 8. In a one-way ANOVA, a significant difference (P < 0.0001) was observed in the zone of inhibition across all fungal strains for each group. To further analyze these differences, a post hoc Dunnett test was performed. The test revealed significant differences (P < 0.001) between all three concentrations of *F. jangomas* (300, 500, 700 μg/disc) and the standard Griseofulvin (50 μg/disc) across all fungal strains. Specifically, the inhibition zones for the *F. jangomas* concentrations were consistently lower than those for the standard Griseofulvin, indicating that the antifungal activity of *F. jangomas* was significantly less potent. This comprehensive analysis suggests that F. jangomas may not have substantial antifungal activity against the tested bacterial strains, as the observed inhibition zones were not comparable to those produced by the standard antifungal agent Griseofulvin.

**Table 8:** Diameter of zone of inhibition of methanol extract of *F. jangomas.*

Sample	Conc. (μg/mL)	Diameter of Zone of Inhibition (mm)			
		Aspergillus niger	Penicillium chrysogenum	*Saccharomyces cerevisiae*	Mucor hiemalis
**Standard (Griseofulvin)**	**50**	20	19	20	21
**FJ**	**300**	8	6	6	7
	**500**	9	6	11	8
	**700**	12	10	12	15
**Blank**	**10**	6	7	7	6

### 3.7 Membrane stabilization

Based on the information provided in Table 9, the methanol extract of this plant demonstrated a noteworthy ability to protect the human erythrocyte membrane from heat-induced lysis. This was observed at a concentration of 500 μg/mL, as indicated by the substantial percentage of inhibitions of hemolysis. Nevertheless, the extract concentrations showed higher % lysis inhibitions than those achieved with 500 μg/mL of Diclofenac Sodium. The precise quantitative measure of the impact of the methanol extract of *F. jangomas* on the destruction of red blood cell membranes caused by heat is recorded in Table 9.

**Table 9:** Effect of MEFJ on heat-induced hemolysis of RBC membrane.

Conc. (μg/mL)	Absorbance Of Sample		Absorbance of control (Distilled water)	%Hemolysis= (OD of test/OD of control)∗100	%Protection = 100-(%Hemolysis)
500	FJ	0.173	0.990	17.47	82.52
	Absorb. of Standard (Diclofenac Sodium)	0.261		26.36	73.63

### 3.8 Screening of analgesic activity

#### 3.8.1 Analgesic activity test by acetic acid-induced writhing method

The study investigated the impact of FJ extract on acetic acid-induced abdominal constrictions in mice, and the results are presented in Table 10. Significantly, the extract was administered at different levels (200 and 400 mg/kg), and the reference medicine, Diclofenac-Na, was given at 10 mg/kg. Both the extract and the drug caused significant reductions in abdominal writhing compared to the negative control group. The considerable reduction resulted in a substantial drop in the mean number of writhing events from 10.78 ± 1.14 in the negative control group to 3.2 ± 0.33 at the 400 mg/kg extract dose. Significantly, the decrease that was seen followed a clear pattern where the effectiveness of the treatment was directly related to the dosage administered. In addition, the extract demonstrated a correlation between the dose administered and the increase in the suppression of abdominal writhing. The inhibition rates ranged from 0 % in the negative control group to 70.32 % at the 400 mg/kg dose. This significant rise in inhibition highlights the correlation between the dosage of the extract and its efficacy in reducing acetic acid-induced abdominal constrictions in mice. The results emphasize the potential therapeutic importance of FJ extract as a pain-relieving substance. This justifies the need for additional research on its mechanisms of action and practical use in clinical settings.

**Table 10:** Analgesic activity FJ on mice by writhing test.

Group	Average Body Weight of Mice (gm.)	Writhing Counting					Mean	SD	SEM	% Writhing	% of Inhibition
		M 1	M2	M3	M4	M5					
**Control**	22–27	12	13	13	7	11	10.78	2.29	1.14	100	0.00
**Positive control**		3	2	3	1	2	2.2	0.74	0.33	20.40	79.6
**Group-1 (400)**		2	3	4	3	4	3.2	0.74	0.33	29.68	70.32
**Group-2 (200)**		5	2	3	7	3	4	1.7	0.8	37.10	62.89

#### 3.8.2 Analgesic activity by hot plate test

The analysis results on the impact of FJ using the hot plate method are presented in Table 11. Initially, there was no significant difference in Pain Reaction Time during the pre-drug testing phase. However, after administering the drug and extract, there were notable changes in Pain Reaction Time when comparing the pre and post-drug phases. Both the reference drug Diclofenac-Na (10 mg/kg) and the extract, given at doses of 200 and 400 mg/kg, showed a significant increase in Pain Reaction Time. Interestingly, the extract at the 200 mg/kg dose had a more substantial effect compared to both the reference drug and the 400 mg/kg dose of the extract. These findings suggest that the extract, particularly at the 200 mg/kg dose, may have potential analgesic properties and should be further investigated.

**Table 11:** Data table for hot plate test for plant extract of FJ.

Group	Time	Mean Tolerance Time (sec)	SD	SE
**Control**	**0min**	6.4	2.24	1.00
	**30min**	7.6	1.85	0.82
	**60min**	06	2.28	1.01
	**90min**	5.4	2.15	0.96
**Positive Control** (Diclofenac-Na, 10 mg/kg)	**0min**	8.6	2.41	1.08
	**30min**	9.4	7.68	3.43
	**60min**	11	8.98	4.01
	**90min**	6.6	8.08	3.61
**400 mg/kg**	**0min**	6.6	1.85	0.829
	**30min**	17.2	2.71	1.21
	**60min**	3.8	7.6	3.39
	**90min**	7.4	9.06	4.05
**200 mg/kg**	**0min**	9.2	2.22	0.99
	**30min**	12	2.68	1.2
	**60min**	9.4	5.57	2.49
	**90min**	11.6	4.02	1.80

## 4 Conclusion

The future for medicinal plants is promising, with over half a million plant species worldwide, many of which remain unexplored for their therapeutic potential. This study highlights the significance of *Flacourtia jangomas* as a valuable source of bioactive compounds, underscoring the necessity for more detailed research. The methanolic extract of *Flacourtia jangomas* bark exhibited a range of biological activities, including antioxidant, cytotoxic, thrombolytic, antibacterial, membrane stabilizing, and analgesic properties. Phytochemical screening revealed the presence of reducing sugars, tannins, saponins, alkaloids, glycosides, and flavonoids, alongside a notable phenolic content. The extract demonstrated antioxidant activity comparable to tert-butylhydroxytoluene (BHT) in DPPH assays. Bioassay tests indicated significant cytotoxicity in brine shrimp, while the clot lysis method showed a positive correlation between extract concentration and thrombolytic efficacy. Antibacterial activity was pronounced at higher concentrations, and membrane stabilizing activity surpassed standard protection rates in heat-induced hemolysis assays. Analgesic effects were confirmed through both the writhing and hot plate tests. These findings suggest that *Flacourtia jangomas* has significant potential as a source of bioactive compounds. Further research is essential to isolate and characterize these components, which may contribute to the development of novel therapeutic agents.

## CRediT authorship contribution statement

**Sadia Afreen Chowdhury:** Writing – original draft, Resources, Methodology, Investigation, Formal analysis, Conceptualization. **Rajib Das:** Writing – original draft, Methodology, Investigation, Formal analysis. **Md. Ariful Haq:** Investigation. **A.T.M. Zafrul Azam:** Writing – review & editing, Supervision. **Choudhury Mahmood Hasan:** Writing – review & editing, Formal analysis. **Monira Ahsan:** Writing – review & editing, Supervision, Formal analysis, Conceptualization.

## Ethics statement

This study was conducted following the guidelines established by the Ethical Review Committee of the Faculty of Sciences, Stamford University Bangladesh. The study was reviewed and approved by the ethics committee prior to the commencement of the experiments, and the ethics approval number is SUB/SF/EC-2402/03. All experimental procedures were conducted in strict accordance with relevant national and institutional guidelines for the care and use of laboratory animals.

## Data availability statement

The data will be available on request.

## Funding

This research received no external funding.

## Declaration of competing interest

The authors declare that they have no known competing financial interests or personal relationships that could have appeared to influence the work reported in this paper.
