# Supplementary material for: Isolation of methyl caffeate and flacourtin from Flacourtia jangomas with comprehensive in-vitro and in-vivo pharmacological evaluation
Source: Heliyon. 2024 Nov 16;10(23):e40445. doi: 10.1016/j.heliyon.2024.e40445 (PMC11625119; doi:10.1016/j.heliyon.2024.e40445)
Supplement: Multimedia component 2 [file mmc2.pdf]

IBSPS, BCSIR, 1H NMR of FJC-140 in CD3OD, Sadia Afreen

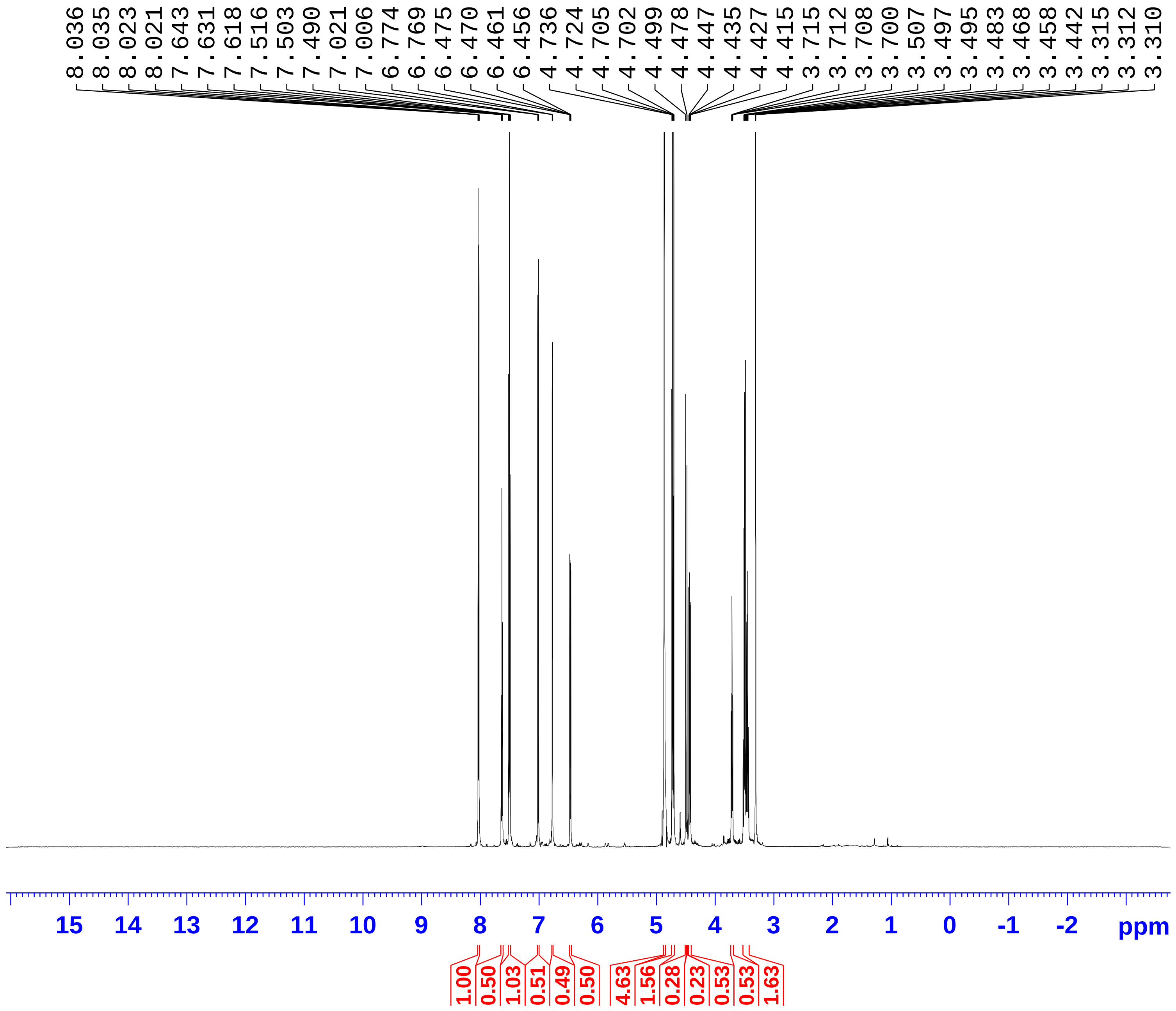

BRUKER

Current Data Parameters

NAME	Dr. Munira Ahsan
EXPNO	5
PROCNO	1

F2 - Acquisition Parameters

Date_	20230620
Time	11.23 h
INSTRUM	BCSIR-600MHz-448614
PROBHD	Z154705_0118 (
PULPROG	zg30
TD	65536
SOLVENT	MeOD
NS	16
DS	2
SWH	11904.762 Hz
FIDRES	0.363304 Hz
AQ	2.7525120 sec
RG	101
DW	42.000 usec
DE	8.79 usec
TE	297.0 K
D1	1.00000000 sec
TD0	1
SFO1	600.3037069 MHz
NUC1	1H
P0	3.33 usec
P1	10.00 usec
PLW1	21.07600021 W

F2 - Processing parameters

SI	65536
SF	600.3000109 MHz
WDW	EM
SSB	0
LB	0.30 Hz
GB	0
PC	1.00

IBSPS, BCSIR, 1H NMR of FJC-140 in CD3OD, Sadia Afreen

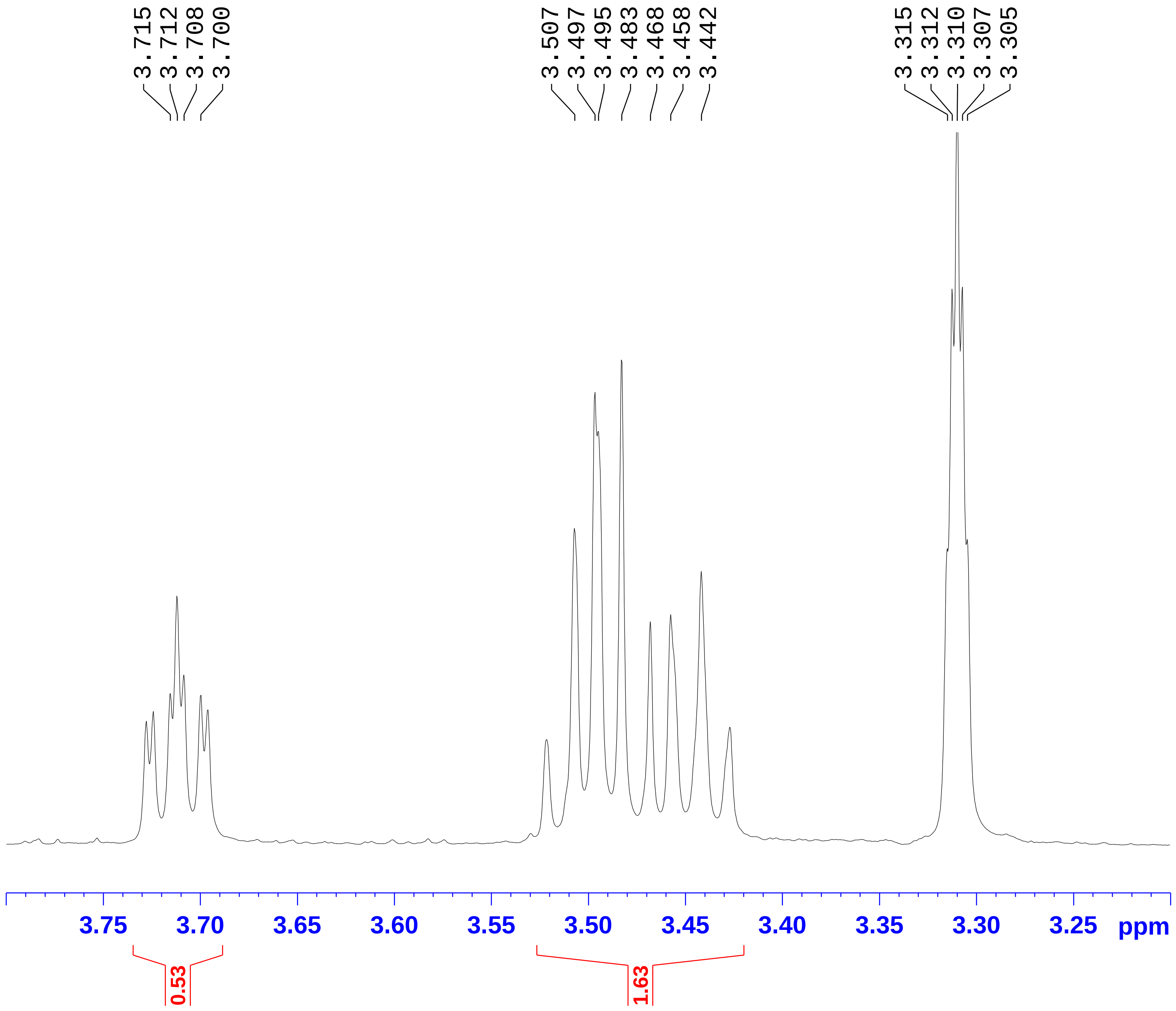

Current Data Parameters
NAME Dr. Munira Ahsan
EXPNO 5
PROCNO 1

F2 - Acquisition Parameters
Date_ 20230620
Time 11.23 h
INSTRUM BCSIR-600MHz-448614
PROBHD Z154705_0118 (
PULPROG zg30
TD 65536
SOLVENT MeOD
NS 16
DS 2
SWH 11904.762 Hz
FIDRES 0.363304 Hz
AQ 2.7525120 sec
RG 101
DW 42.000 usec
DE 8.79 usec
TE 297.0 K
D1 1.00000000 sec
TD0 1
SFO1 600.3037069 MHz
NUC1 1H
P0 3.33 usec
P1 10.00 usec
PLW1 21.07600021 W

F2 - Processing parameters
SI 65536
SF 600.3000109 MHz
WDW EM
SSB 0
LB 0.30 Hz
GB 0
PC 1.00

IBSPS, BCSIR, 1H NMR of FJC-140 in CD3OD, Sadia Afreen

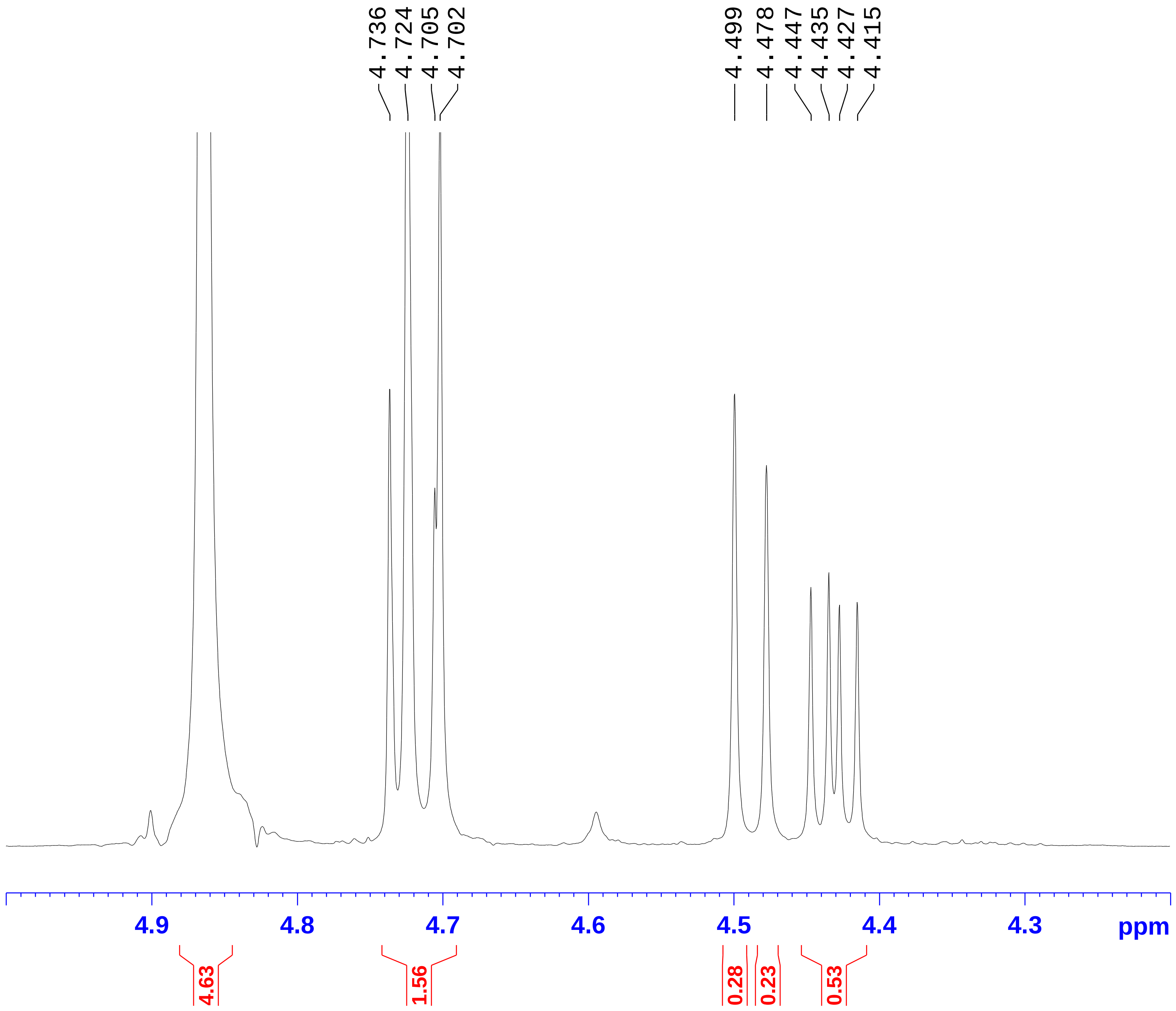

BRUKER

Current Data Parameters

NAME	Dr. Munira Ahsan
EXPNO	5
PROCNO	1

F2 - Acquisition Parameters

Date_	20230620
Time	11.23 h
INSTRUM	BCSIR-600MHz-448614
PROBHD	Z154705_0118 (
PULPROG	zg30
TD	65536
SOLVENT	MeOD
NS	16
DS	2
SWH	11904.762 Hz
FIDRES	0.363304 Hz
AQ	2.7525120 sec
RG	101
DW	42.000 usec
DE	8.79 usec
TE	297.0 K
D1	1.00000000 sec
TD0	1
SFO1	600.3037069 MHz
NUC1	1H
P0	3.33 usec
P1	10.00 usec
PLW1	21.07600021 W

F2 - Processing parameters

SI	65536
SF	600.3000109 MHz
WDW	EM
SSB	0
LB	0.30 Hz
GB	0
PC	1.00

IBSPS, BCSIR, 1H NMR of FJC-140 in CD3OD, Sadia Afreen

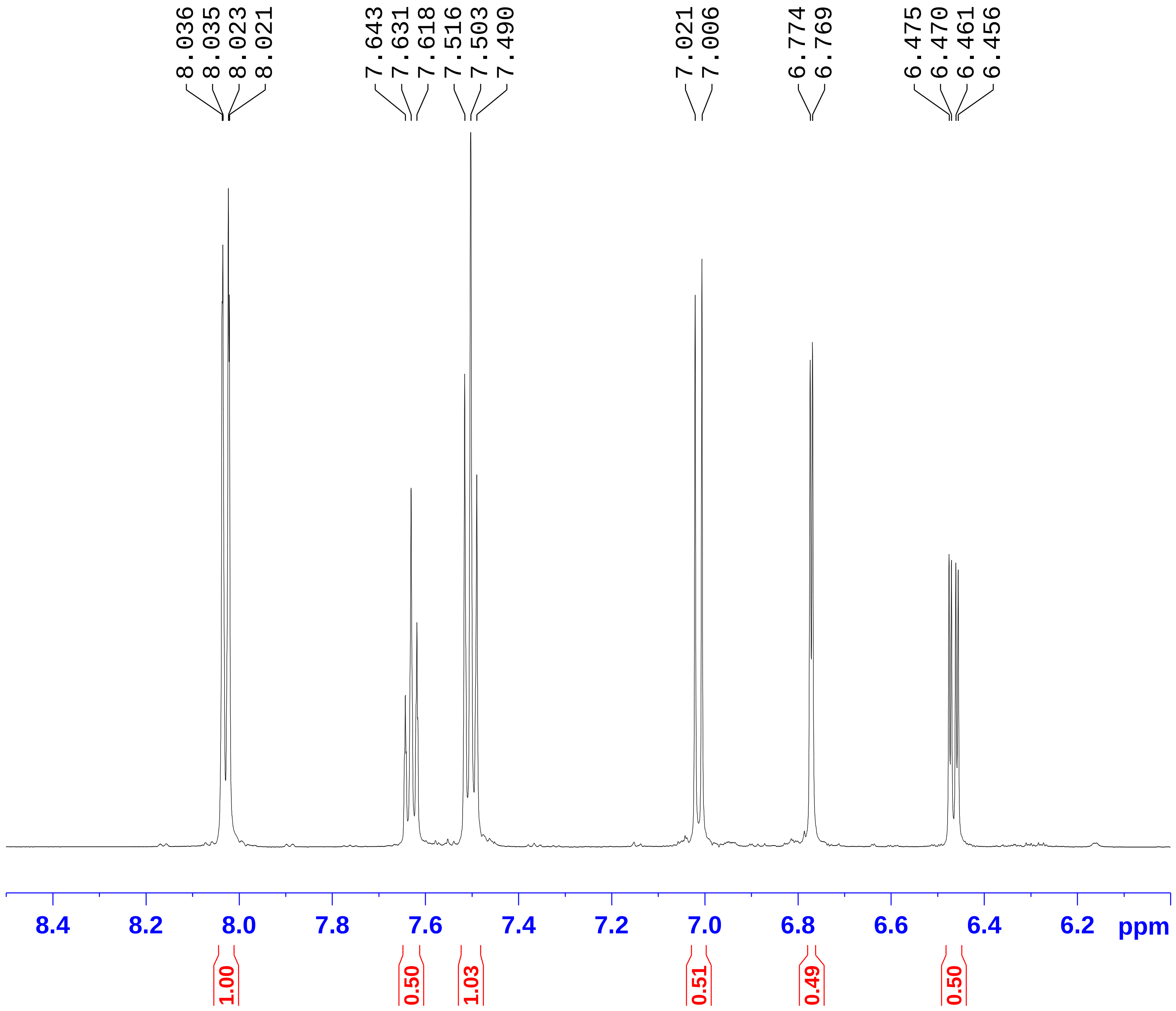

Current Data Parameters

NAME	Dr. Munira Ahsan
EXPNO	5
PROCNO	1

F2 - Acquisition Parameters

Date_	20230620
Time	11.23 h
INSTRUM	BCSIR-600MHz-448614
PROBHD	Z154705_0118 (
PULPROG	zg30
TD	65536
SOLVENT	MeOD
NS	16
DS	2
SWH	11904.762 Hz
FIDRES	0.363304 Hz
AQ	2.7525120 sec
RG	101
DW	42.000 usec
DE	8.79 usec
TE	297.0 K
D1	1.00000000 sec
TD0	1
SFO1	600.3037069 MHz
NUC1	1H
P0	3.33 usec
P1	10.00 usec
PLW1	21.07600021 W

F2 - Processing parameters

SI	65536
SF	600.3000109 MHz
WDW	EM
SSB	0
LB	0.30 Hz
GB	0
PC	1.00
