# Supplementary material for: Isolation of methyl caffeate and flacourtin from Flacourtia jangomas with comprehensive in-vitro and in-vivo pharmacological evaluation
Source: Heliyon. 2024 Nov 16;10(23):e40445. doi: 10.1016/j.heliyon.2024.e40445 (PMC11625119; doi:10.1016/j.heliyon.2024.e40445)
Supplement: Multimedia component 3 [file mmc3.docx]

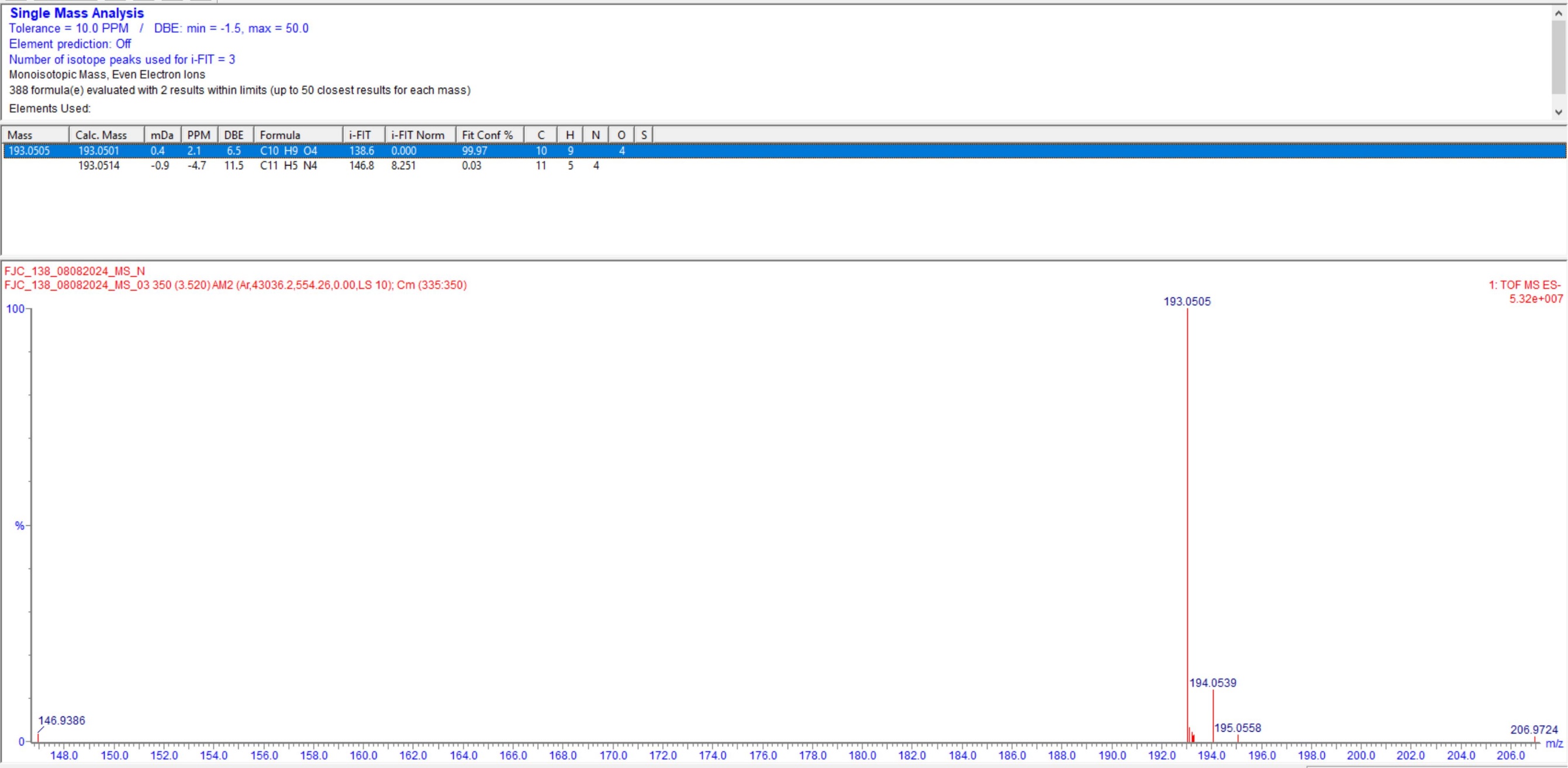


**Figure 1: Mass Spectrum of Compound 1 (FJC-138)-Methyl caffeate.**

**
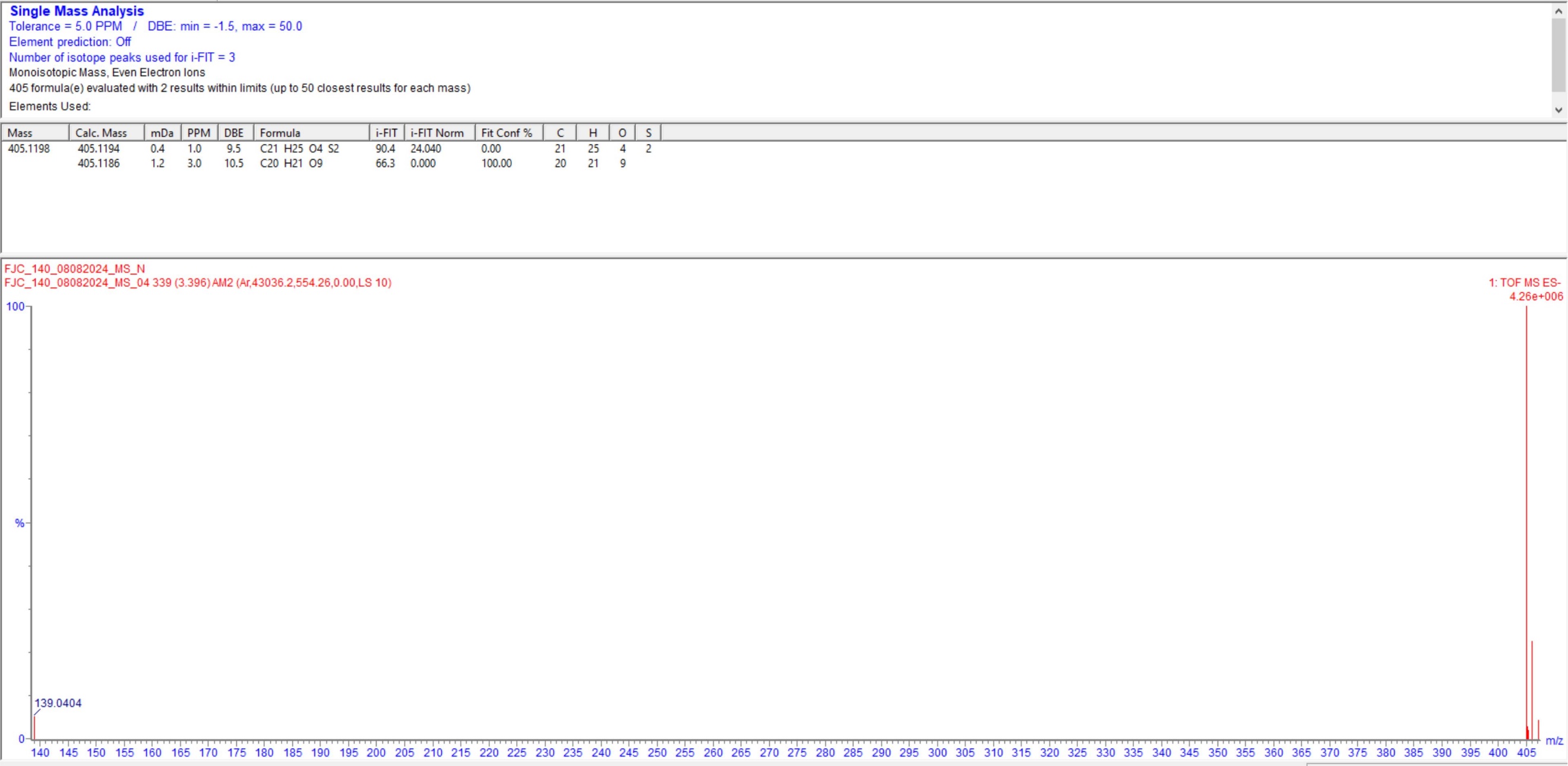
**

**Figure 2: Mass Spectrum of Compound 2 (FJC-140)- Flacourtin.**
