# Supplementary figures and images for: Isolation of methyl caffeate and flacourtin from Flacourtia jangomas with comprehensive in-vitro and in-vivo pharmacological evaluation
Source: Heliyon. 2024 Nov 16;10(23):e40445. doi: 10.1016/j.heliyon.2024.e40445 (PMC11625119; doi:10.1016/j.heliyon.2024.e40445)

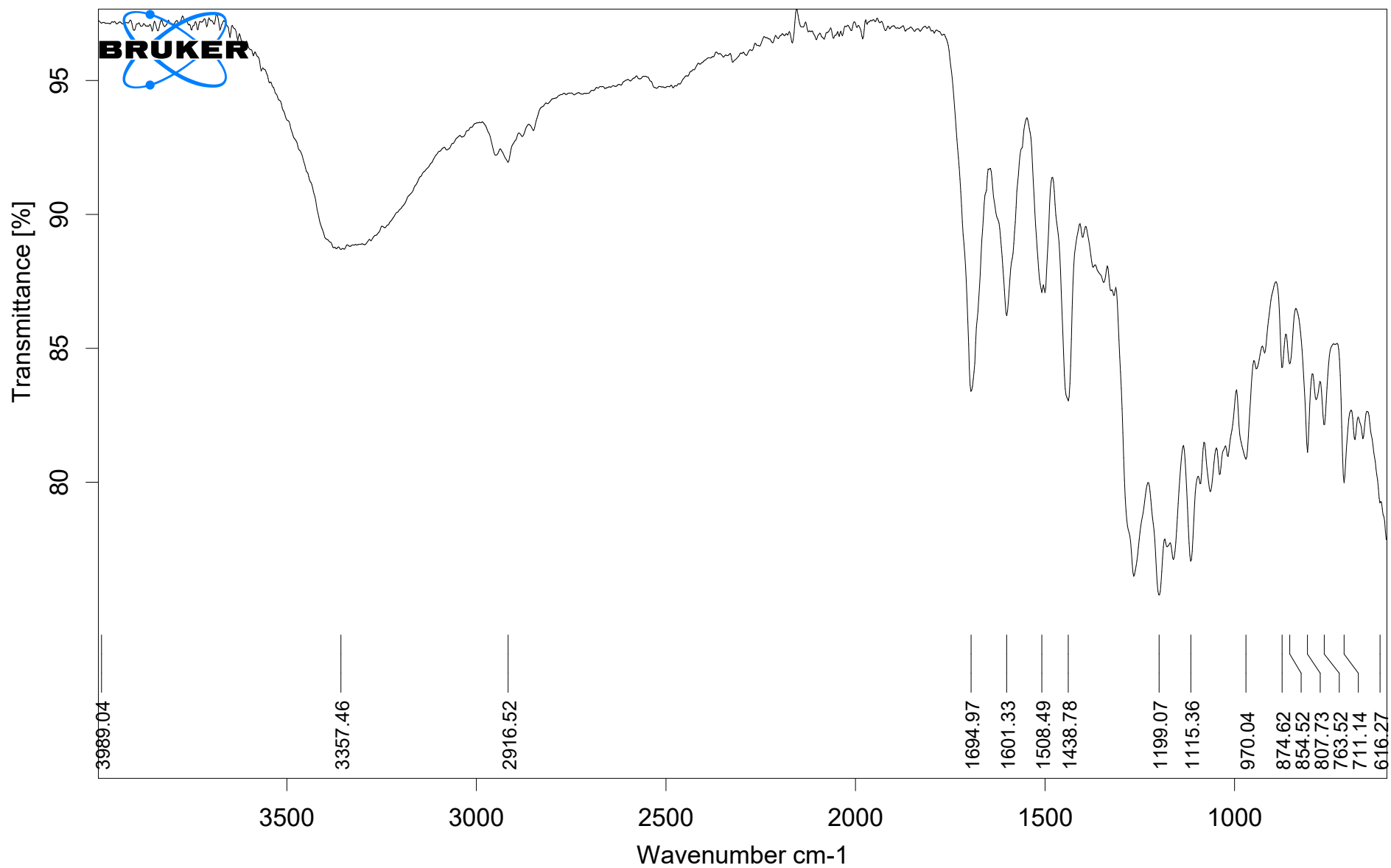

E:\BSPS\_BCSIR\ATR\ATR\_DATA\FJC 138.0

FJC 138

Instrument type and / or accessory

8/4/2024

Supplement: Multimedia component 5 [file mmc5.pdf]

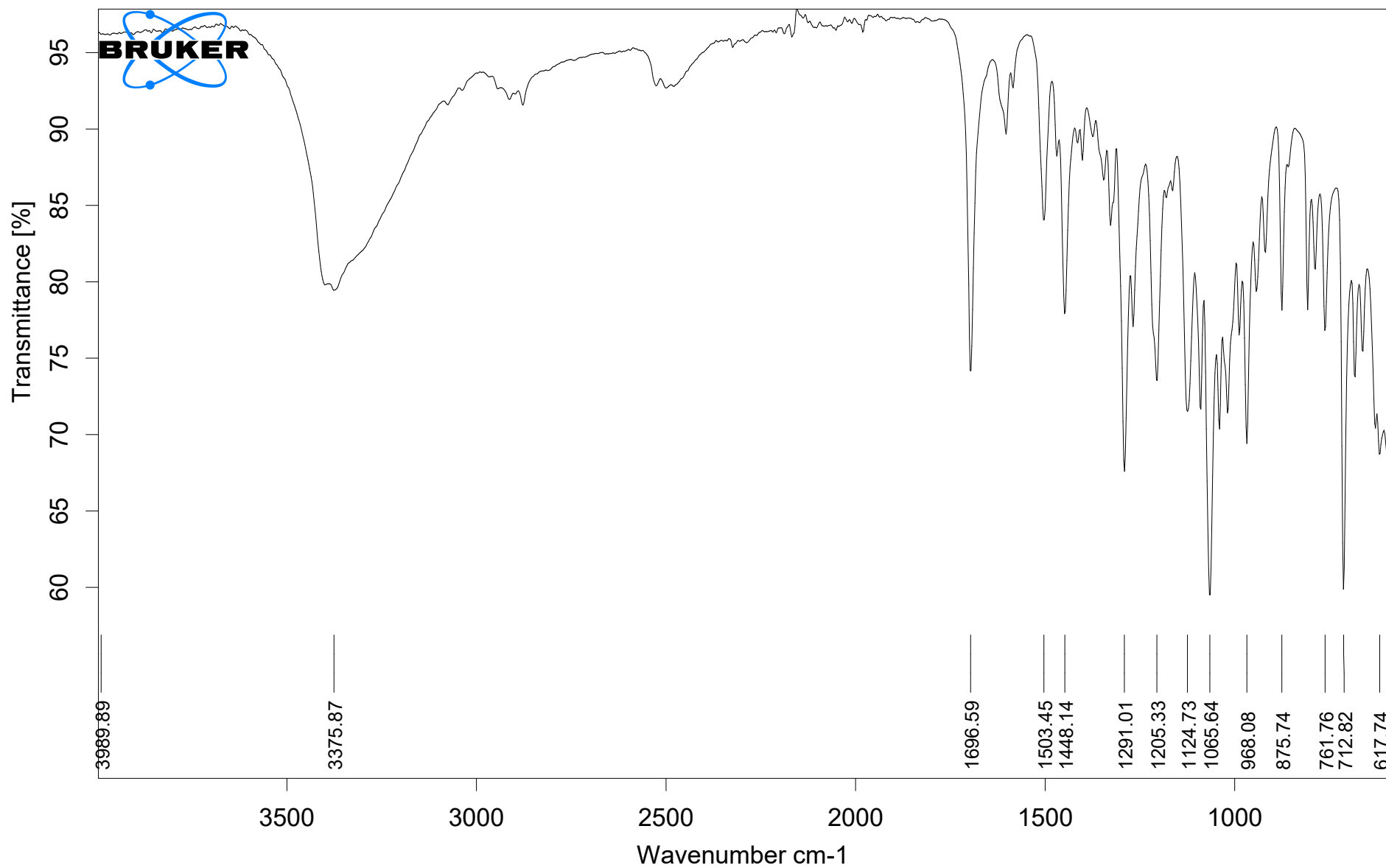

E:\BSPS\_BCSIR\ATR\ATR\_DATA\FJC 140.0

FJC 140

Instrument type and / or accessory

8/4/2024

Supplement: Multimedia component 6 [file mmc6.pdf]
